# Injury pattern of feet and lower limbs in a feet-first fall from height

**DOI:** 10.1007/s12024-025-00939-3

**Published:** 2025-01-09

**Authors:** Larissa Amadasi, Alberto Amadasi, Claas Buschmann, Michael Tsokos

**Affiliations:** 1 Institute of Legal Medicine and Forensic Sciences, Charité - Universitätsmedizin Berlin, Turmstrasse 21, Berlin, 10559 Germany; 2 Institute of Legal Medicine, University Hospital Schleswig-Holstein, Kiel/Lübeck, Germany; 3 State Institute of Legal Medicine, Turmstrasse 21, Berlin, 10559 Germany

**Keywords:** Autopsy, Fall from height, Feet-first fall, Lower limbs fractures, Comminuted fractures

## Abstract

In fatalities caused by falls from height, the analysis of the injury pattern, alongside with circumstantial data, is crucial for understanding the dynamics of the incident. In rare cases, even a differentiation between accidental and intentional events might be possible. The injury pattern of the lower limbs is particularly significant in this context. In the present case - fall from height with primary impact on the feet - fractures of the lower limbs were diagnosed, including comminuted fractures in the middle-distal third of the tibiae and bilateral perforation of both feet and shoes by bone fragments. The longitudinal transmission of high kinetic energy caused by the impact could thus be proven. This specific feature allowed to conduct a complete and accurate assessment of injuries resulting from falls from height.

## Introduction

Falls from height are not rare in forensic routine, often resulting in severe injuries or death. The respective injury pattern can provide crucial insights into dynamics and circumstances surrounding the incident. The injury pattern is influenced by factors such as the height of the fall, the surface onto which the individual fell, and the position of the body upon impact [1–4]. Differentiation between accidental or suicidal falls based on injury patterns is a critical aspect of forensic analysis since injuries may reflect the trajectory and impact surfaces on the body [5–8]. Investigators also consider circumstantial evidence, such as the individual’s mental health history, which can aid in establishing intent. In the forensic literature, the aspect of injuries to the lower limbs in the case of falls on feet from great heights has not yet been described in detail. Ultimately, a comprehensive examination of the injuries at autopsy, combined with scene analysis and contextual factors, is essential in determining the nature of the fall.

## Case report

A 58-year-old man climbed a banister on the fifth floor of a crowded shopping centre and fell about 35–40 m before hitting the ground (concrete covered with ceramic tiles). The incident was observed by several witnesses who reported that the man had fallen in an “upright” position, hitting the ground with his feet and then “bending over”. Resuscitation efforts were not attempted, and the man was pronounced dead on the scene. A suicide note was found in his room at a drug help centre. He had suffered from hebephrenic schizophrenia, but had refused to take medication.

The victim was 174 cm tall and weighed 88 kg, with a BMI of 29.1 kg/m^2^ indicating overweight. Prior to autopsy a full-body CT scan was performed. The autopsy showed a fatal polytrauma (severe chest trauma with lacerations of the lungs, pericardium and heart as well as the ascending thoracic aorta and subsequent bilateral pneumo- and haemothorax, several ruptures of the spleen, of the transverse colon and the right kidney and gross disruption of the pelvic ring). Furthermore, a fracture of the left femur at the condyles was present.

**Fig. 1:**
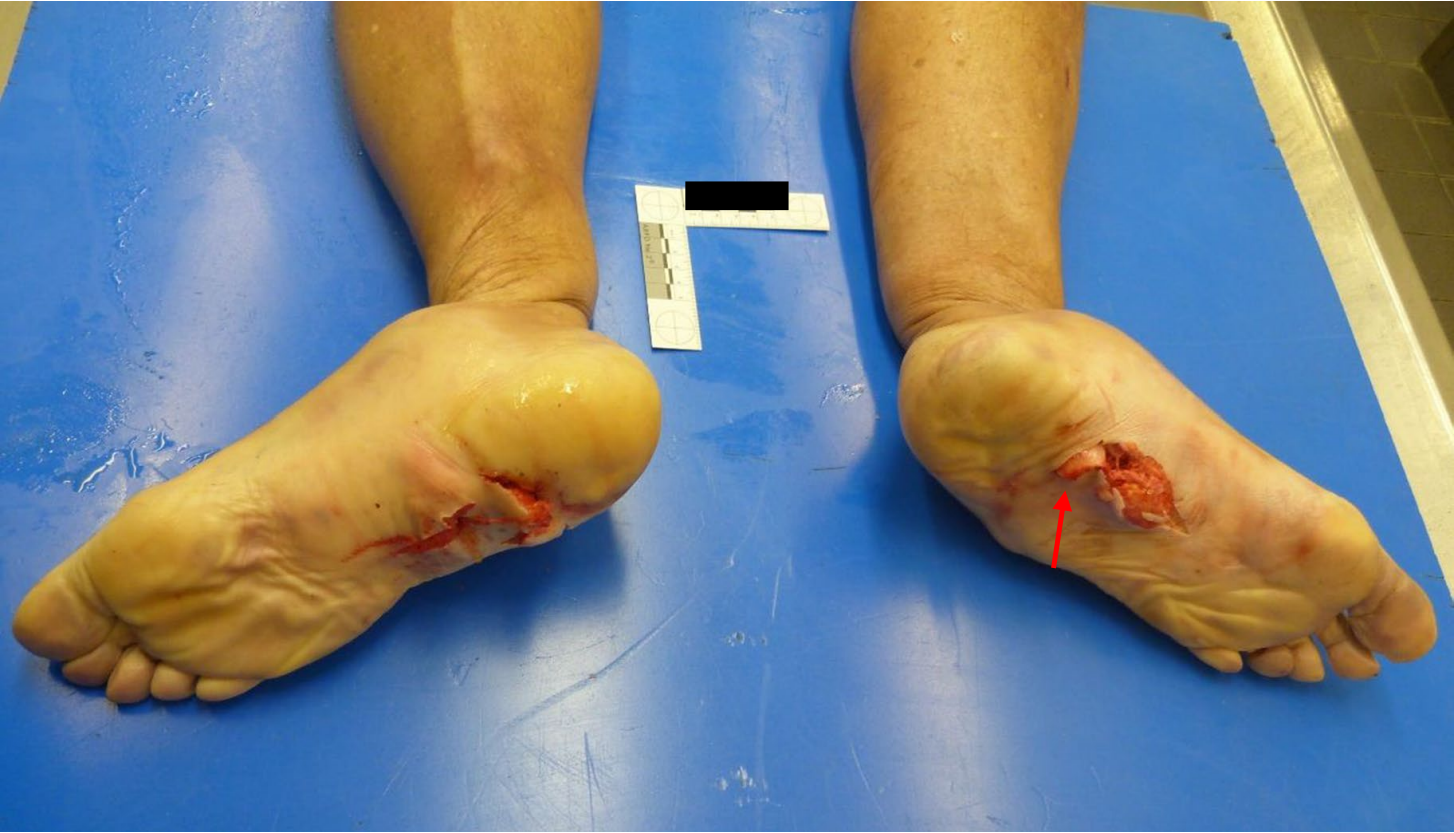
Irregular “stellate” lesions on both feet soles with fragment of the distal part of the tibia within the lesion on the left foot (red arrow)

**Fig. 2:**
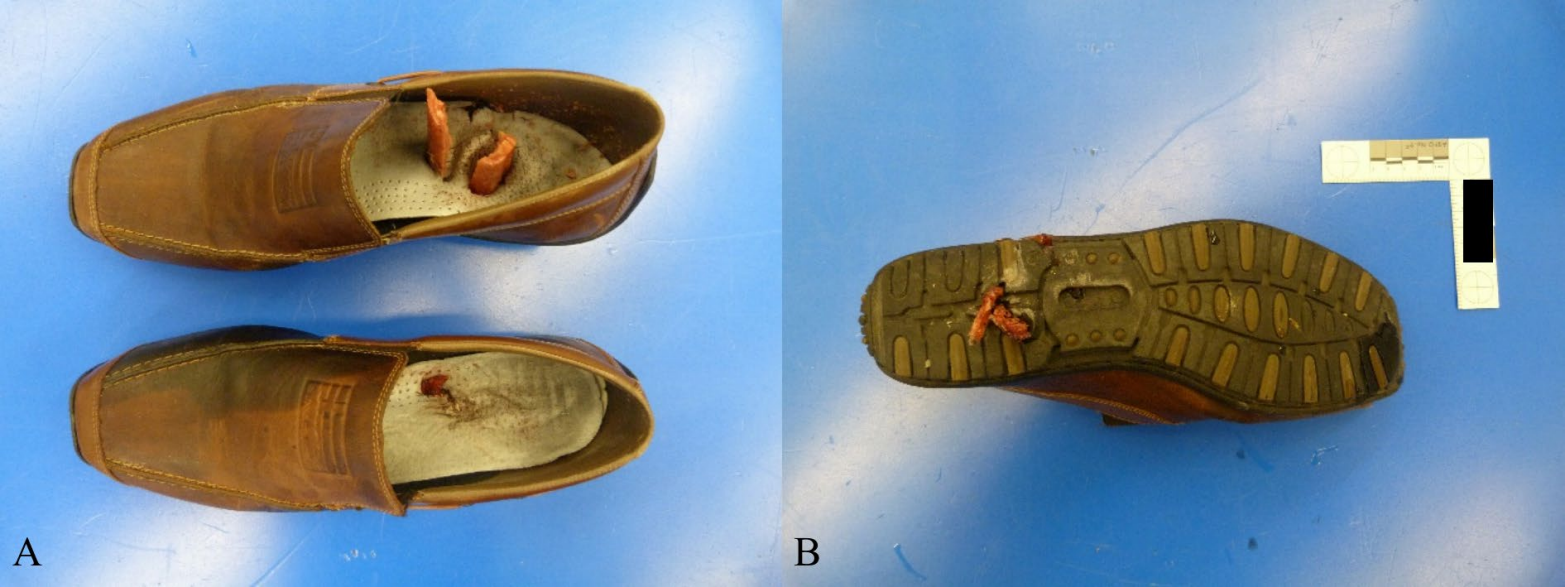
View of the inside of the shoes with fragments of tibia bones embedded in the sole (**A**) and also penetrating the bottom of the sole on the right side (**B**)

**Fig. 3:**
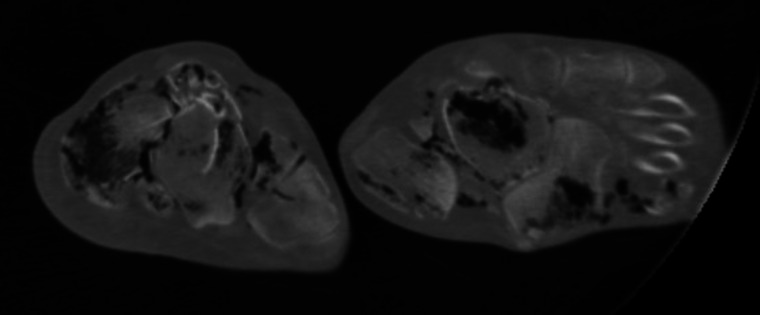
CT images showing comminuted diffuse fractures of the feet bones

**Fig. 4:**
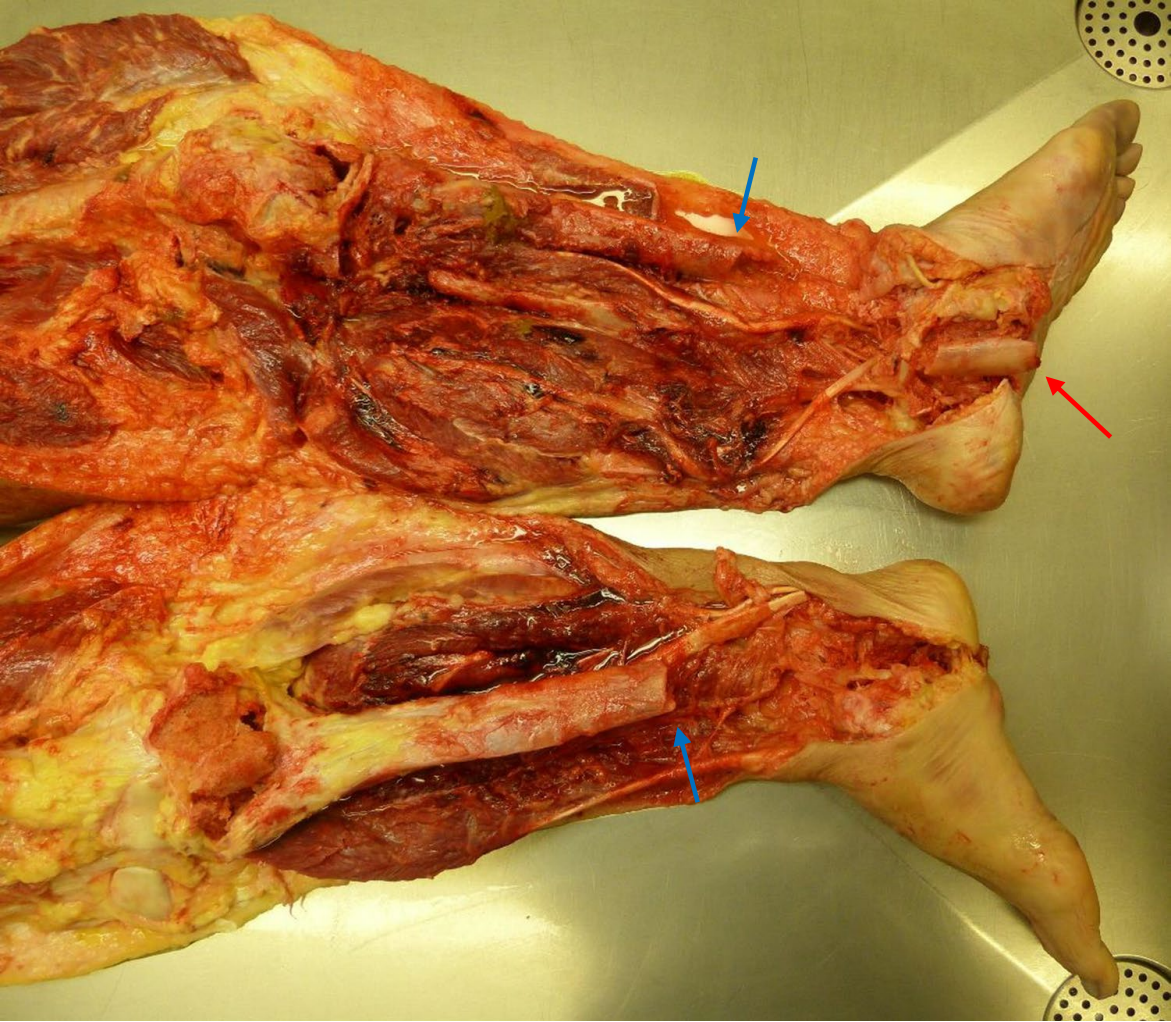
Complete mid-distal third tibia fracture (blue arrows) with comminuted fractures of the distal third (fragments removed in the photo). Fragment of the left tibia embedded in the skin also shown in Fig. 1 (red arrow)

A peculiar aspect was observed in the lower limbs: bilateral comminuted fractures of tibia, fibula and feet bones with vertical perforation of the shoes in the sole area by shin bone splinters. Both soles of the feet showed irregular “star-shaped” lacerations with outward extrusion of soft tissue and partial presence of bone fragments of the tibia (Fig. 1). Both soles of the shoes were penetrated by fragments of the tibia, on the right with complete penetration of the sole by two bone fragments (Fig. 2). The soles were approximately 2.5 cm thick and consisted of a leather sole with an underlying rubber structure. Both fibulae and all the feet bones showed several comminuted fractures (Fig. 3). The tibiae showed bilaterally a complete fracture with complete separation of the distal and comminuted bone fragments in the middle-distal third of the shaft. The mid-proximal part of the shaft of both tibiae had no fractures (Fig. 4).

Toxicological test detected concentrations of the benzodiazepine bromazepam indicating a therapeutic intake. No indications of drug misuse in the two months prior to death were found. There was no evidence of alcohol consumption shortly before death, and there was no evidence of impairment of the individual’s ability to act shortly before death. A mild pulmonary emphysema and cirrhosis of the liver were found as pre-existing diseases. Radiological examinations ruled out osteoporosis or other bone diseases.

## Discussion

In forensic routine (poly-) trauma after fall from height is a frequent cause of death, often resulting from either suicide or accidents. Autopsies in these cases establish not only the cause of death but also assist in reconstructing events leading to the impact, including the victim’s position just before hitting the ground [1–8]. Feet-first falls from height with primary impact on the lower extremities lead to significant injuries, including comminuted fractures of the calcaneus (heel bone), tibia, and fibula. The energy transmitted through the legs can also cause injuries to the knees and hips, potentially resulting in fractures or dislocations. Usually, the force from the impact drives bones from the foot into the tibia. The energy from the impact fractures the tibia and usually the fibula [9]. Additionally, the impact leads to soft tissue injuries such as severe contusions, sprains, or tears in ligaments and tendons. The energy may be even sufficient to cause axial loading injuries, leading to vertebral fractures or spinal injuries. The pattern of these injuries can provide critical information about the height of the fall and the position of the individual while hitting the ground which are essential for forensic reconstruction and analysis. The tibia as a weight-bearing bone is susceptible to both diaphyseal (shaft) fractures and intra-articular fractures, particularly at the knee and ankle joints. These injuries can manifest as transverse, oblique, or spiral fractures, depending on the dynamics of the fall and the angle of impact. In addition to fractures, feet-first falls can also result in tibial stress injuries or even tibial plateau fractures.

In the presented case the tibial fractures and their particular appearance indicate the considerable force resulting from the impact along the longitudinal axis of the body, with comminuted fractures at the distal part of the lower limbs and kinetic energy transmitted downwards to the region of the foot, leading to the perforation of the sole of the feet and even the shoes. According to the formula for assessing the speed at the moment of impact, which results from v = √2gh (v = velocity of impact, g = gravitational constant, h = distance), considering a height of between 35 and 40 m, the impact with the ground occurred in this case at a speed of between 26,2 m/s (94,3 km/h) and 28 m/s (100,8 km/h). The kinetic energy that was then transferred to the victim’s body, according to the formula KE = ½ mv^2^, where v is the velocity at impact and m is the mass of the victim, was thus between 29,744 J and 34,496 J [10–12]. Thus, the height of the fall (35–40 m), which resulted in such major injuries, indicating a pattern associated with a feet-first fall and high kinetic energy transmission through a relatively small impact area. Similar injuries have been described in cases of feet-first falls but those found in the presented case provide information about the height of the fall and the respective dynamics. Beyond injuries related to aviation accidents and vehicle collisions [13–16], fatal falls from heights such as buildings, bridges, rock walls, or cliffs are high-velocity events where the body is subject to extreme decelerative forces.
